# Supplementary material for: Causal interplay between lactose intolerance and gut microbiota: a combined bidirectional Mendelian randomization and in vivo validation study
Source: Front Nutr. 2026 Jun 1;13:1803337. doi: 10.3389/fnut.2026.1803337 (PMC13265576; doi:10.3389/fnut.2026.1803337)
Supplement: Supplementary file 1 [file Data_Sheet_1.zip › supplementary materials/Forward/leave one out cross validation/ebi-a-GCST90027670.finngen_R12_E4_LACTONAS.pdf]

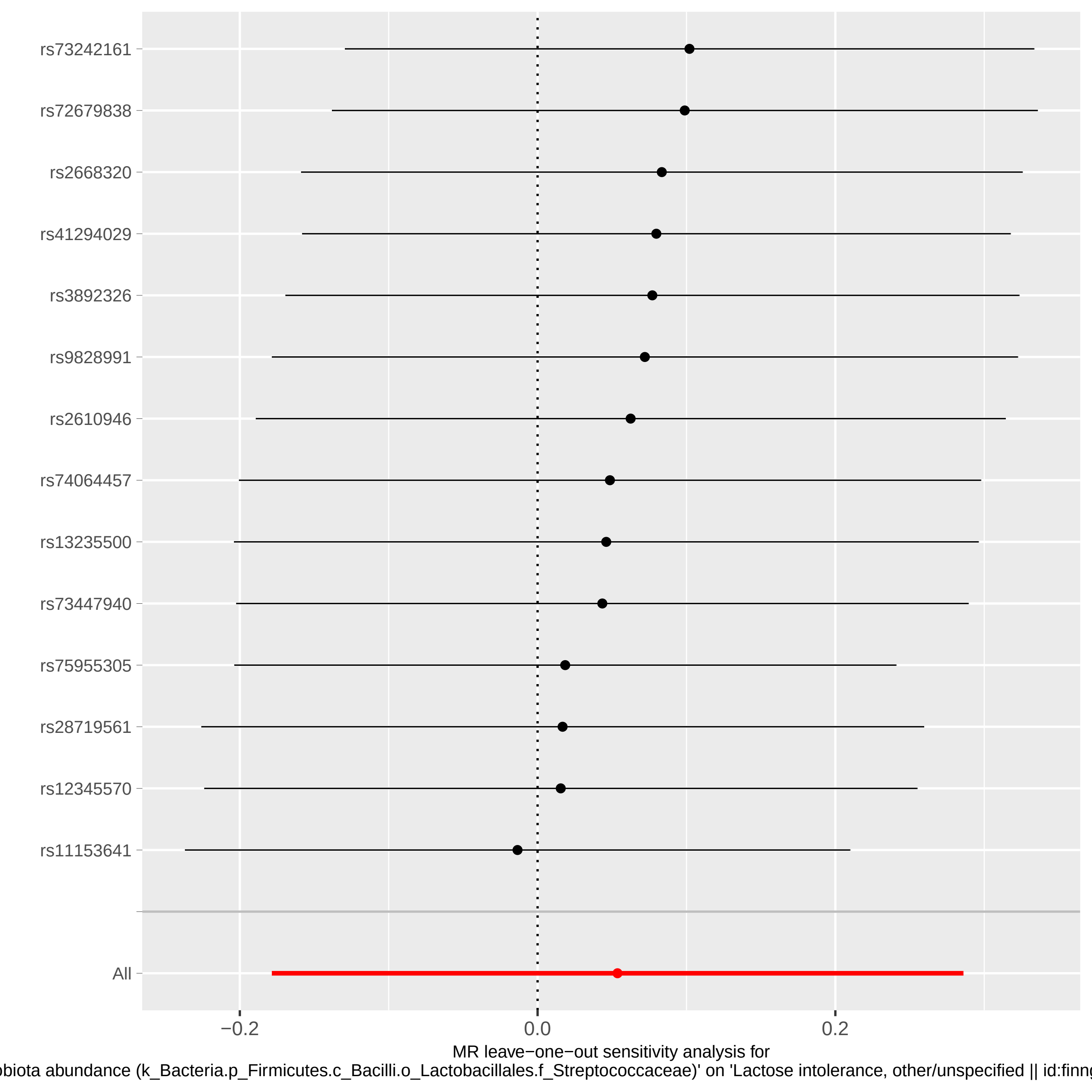
rs73242161
rs72679838
rs2668320
rs41294029
rs3892326
rs9828991
rs2610946
rs74064457
rs13235500
rs73447940
rs75955305
rs28719561
rs12345570
rs11153641
All
−0.2
0.0
0.2
MR leave−one−out sensitivity analysis for
obiota abundance (k_Bacteria.p_Firmicutes.c_Bacilli.o_Lactobacillales.f_Streptococcaceae)' on 'Lactose intolerance, other/unspecified || id:finng
